# Chronic Sodium Selenate Treatment Restores Deficits in Cognition and Synaptic Plasticity in a Murine Model of Tauopathy

**DOI:** 10.3389/fnmol.2020.570223

**Published:** 2020-10-08

**Authors:** Tariq Ahmed, Ann Van der Jeugd, Raphaëlle Caillierez, Luc Buée, David Blum, Rudi D’Hooge, Detlef Balschun

**Affiliations:** ^1^Brain and Cognition, Faculty of Psychology and Educational Sciences, KU Leuven, Leuven, Belgium; ^2^Leuven Brain Institute, Leuven, Belgium; ^3^Laboratory of Biological Psychology, Brain and Cognition, Faculty of Psychology and Educational Sciences, KU Leuven, Leuven, Belgium; ^4^Univ. Lille, Inserm, CHU Lille, U1172—LilNCog—Lille Neuroscience and Cognition, Lille, France; ^5^Alzheimer and Tauopathies, LabEx DISTALZ, Lille, France

**Keywords:** Alzheimer’s disease, chronic oral treatment, synaptic transmission, synaptic plasticity, long-term depression, neurocognitive functions, tau hyperphosphorylation, protein phosphatase 2A (PP2A)

## Abstract

A major goal in diseases is identifying a potential therapeutic agent that is cost-effective and can remedy some, if not all, disease symptoms. In Alzheimer’s disease (AD), aggregation of hyperphosphorylated tau protein is one of the neuropathological hallmarks, and Tau pathology correlates better with cognitive impairments in AD patients than amyloid-β load, supporting a key role of tau-related mechanisms. Selenium is a non-metallic trace element that is incorporated in the brain into selenoproteins. Chronic treatment with sodium selenate, a non-toxic selenium compound, was recently reported to rescue behavioral phenotypes in tau mouse models. Here, we focused on the effects of chronic selenate application on synaptic transmission and synaptic plasticity in THY-Tau22 mice, a transgenic animal model of tauopathies. Three months with a supplement of sodium selenate in the drinking water (12 μg/ml) restored not only impaired neurocognitive functions but also rescued long-term depression (LTD), a major form of synaptic plasticity. Furthermore, selenate reduced the inactive demethylated catalytic subunit of protein phosphatase 2A (PP2A) in THY-Tau22 without affecting total PP2A.Our study provides evidence that chronic dietary selenate rescues functional synaptic deficits of tauopathy and identifies activation of PP2A as the putative mechanism.

## Introduction

Alzheimer’s disease (AD) is a progressive neurodegenerative disorder affecting predominantly brain regions that are required for information processing and memory such as hippocampus and cortex (Squire et al., 2007; Hyman et al., 2012). The phenotypes of AD include deficits in declarative (short- and long-term) memory and disruption in synaptic plasticity, a cellular correlate of memory and undoubtedly one of the first functions to be affected in AD (Selkoe, 2002; Hoover et al., 2010; Takeuchi et al., 2013; Scheltens et al., 2016; Forner et al., 2017). At the neuropathological level, AD is defined by extracellular Aβ plaques, formed as a result of the misprocessing of amyloid precursor protein (APP) as well as aggregation of hyperphosphorylated tau proteins into neurofibrillary tangles (NFTs; Buée et al., 2010; Querfurth and LaFerla, 2010; Masters et al., 2015). The spatiotemporal progression of NFTs from the entorhinal cortex and the hippocampus to the isocortical areas has been reported to correlate well with cognitive deficits and disease progression. Thus, it is Tau pathology and not Aβ-mediated functional deterioration that turned out to correlate better with the severity of dementia (Gomez-Isla et al., 1997; Braak et al., 1998; Nelson et al., 2012; Brier et al., 2016). In accordance with the lower correlation between Aβ-pathology and AD progression, attempts to delay or reverse AD pathology by either removing Aβ or reducing its production had so far not the expected outcome in clinical studies (Lovestone and Manji, 2020; Sabbagh, 2020). It was therefore logical that tau has become the next main target for AD modifying strategies. Apart from the many ongoing Tau-targeting passive and active immunization studies, the diversity of mechanisms that contribute to Tau pathology (e.g., hyperphosphorylation, acetylation, *N*-glycosylation, and truncation) offers multiple targets for therapy development (Congdon and Sigurdsson, 2018). Strategies that target early pathological Tau mechanisms such as Tau hyperphosphorylation and aggregation meet ideally the requirement to shift the time of therapeutic interventions to very early stages of AD. Not only the degree of hyperphosphorylation is decisive for Tau pathology but also the specific pattern of Tau hyperphosphorylation, i.e., which of the many phosphorylations sites of Tau (Sergeant et al., 2008) are (hyper)phosphorylated under the particular pathological condition. These pathological Tau phosphorylation patterns are the result of an intricate balance between the activity of Tau kinases, such as glycogen synthase kinase-3β (GSK3β) as the major Tau kinase, and phosphatases with protein phosphatase 2A (PP2A) as the main representative (Liu et al., 2005; Kremer et al., 2011; Martin et al., 2013). Thus, therapeutic reduction of Tau hyperphosphorylation and subsequent aggregation can be either achieved by inhibition of Tau kinases or activation of phosphatases. Suitable candidate therapeutic compounds should be easy-to-administer, free of toxic side-effects in a broad dose range and show excellent bioavailability and CNS penetrating properties.

All these criteria are met by sodium selenate, an oxidized form of selenium, which was reported to be a selective activator of PP2A (Corcoran et al., 2010b; van Eersel et al., 2010; Cardoso et al., 2019). This is confirmed by a Phase IIa control trial that assessed the safety and tolerability of sodium selenate in forty patients with mild to moderate AD, aged ≥55 years, and found the compound to be safe and well-tolerated (Malpas et al., 2016).

PP2A accounts for ~71% of total tau phosphatase activity in the human brain (Liu et al., 2005); and its expression and/or activity have been found significantly decreased under the conditions of AD pathology (see Lambrecht et al., 2013; Taleski and Sontag, 2018 for further references), which should promote hyperphosphorylation of Tau by disturbing the balance between Tau kinase and phosphatase activities mentioned above. PP2A phosphatases are a diverse family of holoenzymes, comprised of at least two subunits, a scaffolding A subunit and a catalytic C subunit. This dimer can further associate with one of a diverse range (<20) of regulatory B-subunits to form the typical mammalian heterotrimeric holoenzyme. Within these trimeric complexes, the B-type subunits determine important properties like the catalytic activity, substrate specificity, and subcellular localization, and hence, the physiological functions of the holoenzyme (Lambrecht et al., 2013; Taleski and Sontag, 2018). Important to mention, the predominant neuronal PP2A holoenzyme that shows the highest tau phosphatase activity and the strongest affinity for tau is the one that contains the Ba (or PPP2R2A or PR55) regulatory subunit (Sontag et al., 1996, 1999; Xu et al., 2008). Important for a candidate therapeutic compound, sodium selenate was found to be non-toxic in primary rat hippocampal slice cultures up to a concentration of 100 μM (Corcoran et al., 2010b; van Eersel et al., 2010), while other selenium compounds such as sodium selenite and selenomethionine showed significant toxicity under the same conditions (Corcoran et al., 2010b; van Eersel et al., 2010).

Here, we examined for the first time the effects of a chronic 3-month dietary application of this compound on the disease phenotype of a tauopathy AD mouse model, THY-Tau22 mice (Schindowski et al., 2006), with the focus on synaptic transmission and plasticity. The selenate treatment started at 9 months, an age at which THY-Tau22 mice progressively develop AD-like hippocampal Tau pathology associated with severe deficits in memory underlined by synaptic impairments (Schindowski et al., 2006; Van der Jeugd et al., 2011, 2013; Burnouf et al., 2013). Our data demonstrate that a chronic dietary supplement with sodium selenate rescues deficits in hippocampal synaptic plasticity and neurocognition in THY-Tau22 mice and disclose an activation of PP2A as the putative underlying mechanism.

## Materials and Methods

### Animals

Twenty male heterozygous transgenic THY-Tau22 mice and a corresponding number of wildtype (WT) sibling controls were used in the present study. THY-Tau22 and WT littermate mice were generated as described previously (Schindowski et al., 2006). THY-Tau22 mice (C57Bl6/J background) overexpress a 1N4R human mutated Tau isoform (G272V and P301S) under a Thy1.2-promoter. All animals were kept in standard animal cages under conventional laboratory conditions (12 h/12 h light-dark cycle, lights on 08:00–20:00, 22°C), with *ad libitum* access to food and water, unless stated otherwise. Behavioral tests were conducted during the light phase of their activity cycle. All behavioral and electrophysiological experiments have been reviewed and approved by the animal experiments committee of the University of Leuven, Belgium, and were carried out following the European Directive 2010/63/EU.

### Chronic Treatment With Sodium Selenate (Na_2_SO_4_)

At the age of 9 months, 12 weeks before the start of experiments, the mice were subdivided into four groups of 10 mice. One group from each genotype was provided with 12 μg/ml sodium selenate in the drinking water (Corcoran et al., 2010b; van Eersel et al., 2010). The other half received normal drinking water. We chose sodium selenate because the closely related inorganic form sodium selenite and the main organic form selenomethionine are toxic in primary hippocampal slice cultures at comparable and even lower concentrations (Corcoran et al., 2010b).

### Time-Line

**Figure 1:**
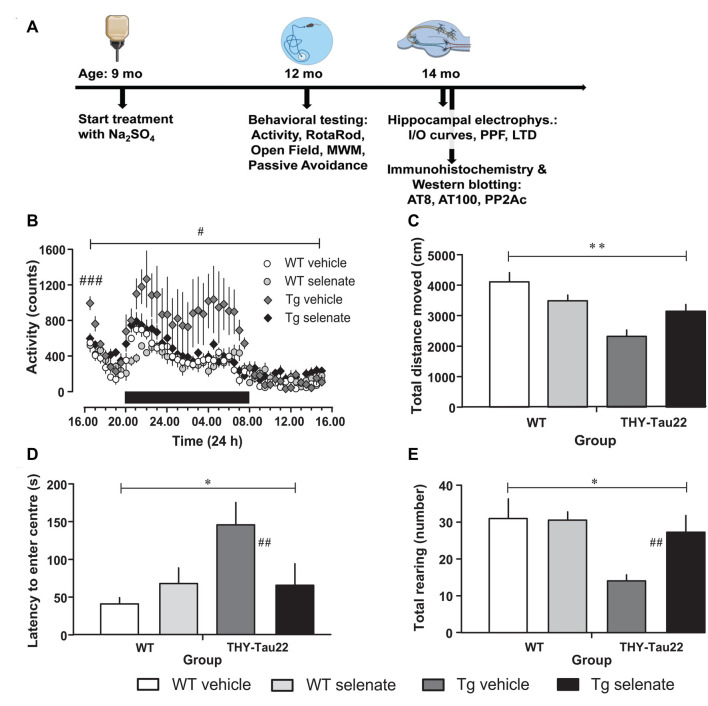
Selenate treatment restores behavioral and neurocognitive deficits in THY-Tau22 (henceforth referred to as Tg) mice to levels comparable with wildtype (WT) controls. **(A)** Timeline of the experiments. The behavioral experiments started at the age of 12 months, the electrophysiological examination at 14 months. At the same time, tissue samples were collected for immunohistochemistry and Western blotting. **(B)** Analysis of cage activity revealed higher activity of Tg mice at the beginning of the recording period and increased nocturnal activity as compared with WT controls. These differences were abolished by chronic selenate treatment. Note that selenate treatment did not affect WT animals. **(C–E)** Open-field behavior. Tg animals traveled less in the arena **(C)** showed higher latencies to enter the center of the arena **(D)** and displayed less exploratory rearings in the center **(E)** as compared with WT mice. Selenate treatment remediated these behavior changes. Mean ± SEM is given; group sizes: cage activity *n* = 10 per group; open field test Tg-veh *n* = 9, Tg-sel *n* = 10, WT-veh *n* = 8, WT-sel *n* = 10. Bar with ^#^ indicates the significance level *p* ≤ 0.05 in RM-ANOVA with a Dunnett *post hoc* test using Tg vehicle as comparison; bars with a * and ** indicate *p* ≤ 0.01 and *p* ≤ 0.001, respectively, in the Two-way ANOVA; ^#^ and ^##^ represent *p* ≤ 0.01 and *p* ≤ 0.001, respectively, in the Dunnett *post hoc* test.

Behavioral testing [Cage Activity, Rotating Rod, Open Field, Morris Water Maze (MWM) and Passive Avoidance] was performed successively as depicted in Figure 1A. Mice were handled for 3 days before the start of the experiments (including weighing the mice and color-coding their tails for further identification). Before each test, animals were habituated to the experimental room for at least 30 min.

### Behavioral Studies

#### Neuromotor Tests

##### Cage Activity

To measure diurnal cage activity, mice were placed individually in 26.7 cm × 20.7 cm transparent cages (floor area 370 cm^2^) that were positioned between three infrared photo beams. Beam crossings that represented locomotor cage activity were registered for each 30-min interval during a 23-h recording period, using an interfaced PC (Goddyn et al., 2006).

##### Rotating Rod

Previous studies on the *Rotating Rod (Rotarod*, MED Associates) documented no motor deficits for THY-Tau22 (Van der Jeugd et al., 2011) but to exclude any effect of the selenate treatment on motor coordination, the rotarod study was repeated.

##### Open Field

Open field exploration was monitored using a brightly lit 50 cm × 50 cm arena. Before the experiment, mice underwent 30 min of dark adaptation inside a dark cupboard. Each mouse was placed in the arena for 10 min. Numbers of entries in the corners and the center (defined as a circle with a 30 cm diameter), the latency of the first corner and center approach, as well as time spent, and velocity in corners and center were measured (Noldus Ethovision, The Netherlands).

#### Neurocognitive Tests

##### Morris Water Maze

Spatial and reference memory abilities were examined in the standard hidden-platform acquisition and retention version of the MWM. Training consisted of five consecutive days of four trials (120 s each) from different starting positions. The time required to locate the hidden escape platform (escape latency in seconds), distance traveled (path length in centimeter), and swimming speed (velocity in cm/s) were recorded (Noldus Ethovision, The Netherlands). After 2 days of rest, a probe trial with the platform removed was performed for 100 s. The time (s) the animals spent in each of the four quadrants, and the frequency the mice swam over the former platform position (n) were measured (Van der Jeugd et al., 2011).

##### Passive Avoidance

Single-trial passive avoidance learning was examined in a step-through box with a small illuminated compartment and a larger dark compartment with a grid-floor. The grid-floor was connected with a constant current shocker (MED Associates Inc., St. Albans, USA). Animals were dark-adapted for 30 min and then placed in the small illuminated compartment. After 5 s, the sliding door to the dark compartment was opened and the entry latency was recorded. When mice entered the dark compartment, a mild foot shock (0.3 mA, 1 s) was delivered. Retention was tested 24 h later according to the same procedure, excepting that animals did not receive a shock. The entry latencies were recorded with a cut-off of 300 s.

### *In vitro* Hippocampal Synaptic Plasticity

After completion of behavioral tests, animals were killed by cervical dislocation and the brain rapidly isolated out into ice-cold (4°C) artificial cerebrospinal fluid (ACSF), oxygen saturated with carbogen (95% O_2_/5% CO_2_). ACSF consisted of (in mM): 124 NaCl, 4.9 KCl, 24.6 NaHCO_3_, 1.20, KH_2_PO_4_, 2.0 CaCl_2_, 2.0 MgCl_2_, 10.0 glucose, pH 7.4. Transverse slices (400 μm thick) were prepared from the dorsal area of the right hippocampus with a tissue chopper and placed into a submerged-type chamber, where they were kept at 32°C and continuously perfused with ACSF at a flow-rate of 2.2 ml/min. After about 90 min incubation, one slice was arbitrarily selected and a lacquer-coated monopolar stainless steel or tungsten electrode was placed in CA1 stratum radiatum for stimulation in constant current mode. For the recording of field excitatory postsynaptic potentials (fEPSPs), a glass electrode (filled with ACSF, 3–7 MΩ) was placed in the stratum radiatum. The time course of the field EPSP was measured as the descending slope function for all sets of experiments. After a further hour of incubation, input/output curves were established and the stimulation strength was adjusted to elicit an fEPSP-slope of 35% of the maximum and was kept constant throughout the experiment. Paired pulse facilitation was investigated by applying two pulses in rapid succession (interpulse intervals of 10, 20, 50, 100, 200, and 500 ms, respectively) at 120 s intervals. During baseline recording, three single stimuli (0.1 ms pulse width; 10 s interval) were measured every 5 min and averaged. LTD was induced by low-frequency stimulation (LFS) consisting of 1,200 pulses at 2 Hz (0.2 ms pulse-width) and repeated three times with a 10-min interval between completion of one LFS-train and the start of the successive one (Balschun et al., 2003; Ahmed et al., 2011, 2015). Immediately after each conditioning stimulus, evoked responses were monitored at 2, 5, and 8 min and then subsequently every 5 min recording up to 4 h after the first conditioning stimulus.

### Immunohistochemistry and Western Blotting

For immunohistological experiments, the tissue was prepared as described in Leboucher et al. (2013). Animals were killed by cervical dislocation, brains were removed, one hemisphere was postfixed for 7 days in 4% paraformaldehyde and then incubated in 20% sucrose for 24 h before being frozen at −40°C in isopentane (methyl butane) and kept frozen at −80°C until use. Free-floating coronal sections (40 μm) were obtained using a cryostat (Leica). The sections of interest were used for free-floating immunohistochemistry using AT8 (Pierce MN-1020, recognizes pS202/T205, 1/200) and AT100 antibodies (Thermo Fisher Scientific, recognizes pT212/pS214; 1/1,000) and finally mounted on Superfrost slides respectively. Of note, although these are mouse IgG1 antibodies, they were proven to work fine in THY-Tau22 mice without causing any disturbing background staining (Leboucher et al., 2013). Staining pixel count was performed blindly by setting the threshold to the same value for each section as previously described using ImageJ software (Scion Software).

The other hemisphere was used for Western blotting as previously described (Laurent et al., 2014). Briefly, tissue was homogenized in 200 μl Tris buffer (pH 7.4) containing 10% sucrose and protease inhibitors (Complete; Roche Diagnostics, Meylan, France), sonicated and kept at −80°C until use. Protein amounts were evaluated using the BCA assay (Pierce, Rockford, IL, USA). Proteins were diluted with lithium dodecyl sulfate buffer (LDS) 2× supplemented with reducing agents (Invitrogen) and then separated on NuPage Novex gels (Invitrogen) for Western blot analysis. Proteins were transferred to nitrocellulose membranes, which were then blocked (5% non-fat dry milk in TNT:Tris-HCl 15 mM, pH 8, NaCl 140 mM, 0.05% Tween) and incubated with primary and secondary antibodies. Signals were visualized using chemiluminescence kits (ECLTM, Amersham Velizy-Villacoublay, Villacoublay, France) and a LAS3000 imaging system (Fujifilm, Tokyo, Japan). We evaluated the expression of the catalytic subunit of PP2A (PP2Ac; Millipore, 1/1,000) as well as PP2Ac demethylation (demethyl PP2Ac; Santa Cruz, 1/1,000) in THY-Tau22 selenate-treated and vehicle-treated mice. We performed two separate Western blots for each antibody using three samples of each group, normalized to GAPDH. The PP2Ac/GAPDH and Demethy-PP2A/GAPDH ratios were calculated as well as the double ratio. PP2A demethylation (Leu 309) was taken as an index of its activity. Indeed, PP2A activity is enhanced by the methylation of its catalytic subunit (PP2Ac) and is conversely decreased by its demethylation (Papon et al., 2013; Sontag and Sontag, 2014).

### Statistical Analysis

Statistical analyses were performed by using IBM SPSS 19 (IBM SPSS, Armonk, NY, USA) and GraphPad Prism 7 (GraphPad Software, La Jolla, CA, USA). Unless otherwise stated, all statistics were done with Two-Way ANOVA or repeated measures ANOVA (RM-ANOVA). For single between-group comparisons, *t*-tests with Welch‘s correction were used.

## Results

### Neuromotor Tests

When cage activity was monitored in the current study, we found a difference between groups (RM-ANOVA over the whole recording time *F*_(3,30)_ = 3.258, *p* = 0.035). *Post hoc* Dunnett tests confirmed that only vehicle-treated THY-Tau22 mice (henceforth referred to as Tg mice for the remainder of the manuscript) demonstrated a significantly different level of exploratory activity compared with age-matched WT mice (*p* = 0.032; Figure 1B). This higher activity level of vehicle-treated Tg mice is not simply due to an increase in the mean activity level but primarily due to a higher initial and nocturnal activity. Noticeably, upon placement, all mice showed increased activity values (when compared to the activity level in the afternoon of the next day), which gradually disappeared. The high initial values and the decay of activity levels during this phase, which we consider as habituation to the novel environment, are apparently different in Tg vehicle-treated mice from the other genotypes as confirmed by a multivariate ANOVA of the first hour of recording (*F*_(3,33)_ = 6.545, *p* = 0.002; *post hoc* Dunnett test vs. vehicle-treated Tg mice, *p* = 0.001). Chronic treatment with sodium selenate corrected for the increased initial and nocturnal activity of THY-Tau22 mice.

Next, we tested general motor abilities and coordination in the rotarod test and found no differences. Thus, all four groups were equally capable of remaining on the rotating rod (*F*_(3,33)_ = 0.747, *p* > 0.05; data not shown). Previous investigations of the ambulatory activity of 10-month-old THY-Tau22 mice had demonstrated an overall increased activity in Tg mice (Van Der Jeugd et al., 2013).

In the Open field, the total path length was significantly different between groups (*F*_(3,32)_ = 7.623, *p* = 0.001; Figure 1C). Vehicle-treated Tg animals tended to travel less distance in the arena compared with treated transgenic animals (*post hoc* Dunnett test *p* = 0.06). Time to approach the center of the arena and number of exploratory rearings in the center were different between groups (overall ANOVA *F*_(3,32)_ = 5.700, *p* = 0.03 for latency to the center and *F*_(3,32)_ = 3.723, *p* = 0.021 for rearing in the center). Non-treated Tg mice stayed longer away from and displayed less exploratory rearings in the center as compared to the vehicle-treated Tg animals (*post hoc* Dunnett tests *p* = 0.007 for center approach latency; Figure 1D and *p* = 0.01 for rearing; Figure 1E). Interestingly, after chronic selenate treatment, these behavioral changes were normalized to the level of WT values.

### Neurocognitive Tests

**Figure 2:**
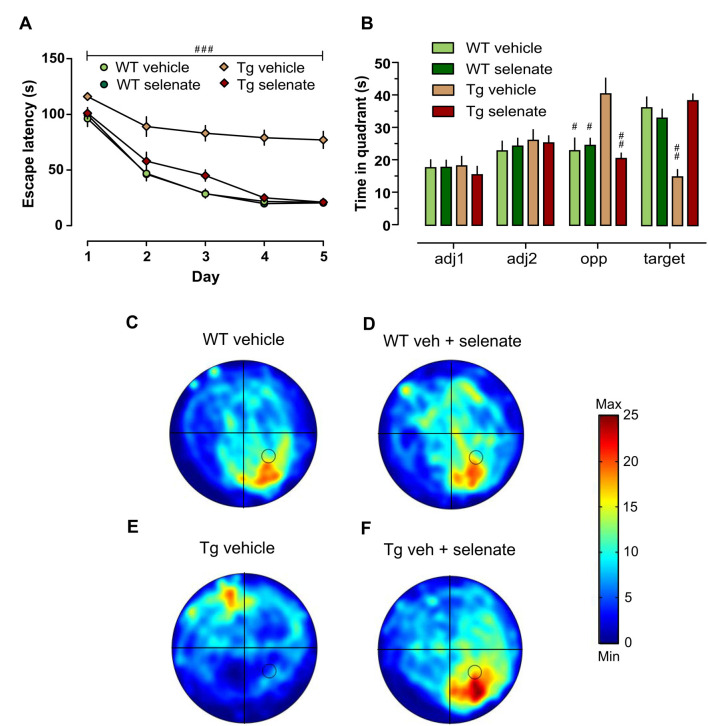
Impaired spatial learning and memory of Tg mice in the Morris water maze (MWM) was restored by treatment with selenate. **(A)** Tg mice provided with dietary selenate displayed escape latencies for locating the hidden platform comparable with the selenate-treated WT and non-treated WT groups. In contrast, the vehicle-treated Tg group were poor learners. **(B)** In the probe test, both WT groups and selenate-treated Tg groups confirmed memory for the platform location by spending significantly more time in the platform quadrant compared to vehicle-treated Tg mice. The vehicle-treated Tg group, in contrast, spent significantly less time in the target quadrant and showed a preference for the opposite quadrant instead (adj = adjacent 1/2, opp = opposite and target; hashes denote statistically significant differences between vehicle-treated Tg mice and the other three groups). **(C–F)** Examples of heat maps of the swim patterns during probe tests. The location of the platform is marked in the heat map as a black circle in the bottom right quadrant. **(C)** WT vehicle, **(D)** WT selenate-treated, **(E)** Tg vehicle, **(F)** Tg selenate-treated. Note the clear target preference of Tg after selenate treatment in panel **(F)** as compared to vehicle-treated Tg in **(E)**. The heat map scale bar indicates the time in seconds. Mean ± SEM is given; group sizes: Tg-veh *n* = 9, Tg-sel *n* = 10, WT-veh *n* = 8, WT-sel *n* = 9. Bar with ^###^ indicates significance level *p* ≤ 0.001 of RM-ANOVA with a Dunnett *post hoc* test using Tg vehicle as comparison; ^#^ and ^##^ represent *p* ≤ 0.01 and *p* ≤ 0.001, respectively, in the Dunnett *post hoc* test.

We assessed spatial learning and memory in the established hippocampus-dependent water maze paradigm. Looking at both the time to locate the hidden platform and the path length traveled to reach it, we found significant differences between the groups (overall RM-ANOVA *F*_(3,33)_ = 44.542, *p* = 0.0001 for escape latency and *F*_(3,33)_ = 43.466, *p* = 0.0001 for distance traveled). *Post hoc* Dunnett tests revealed that spatial acquisition in vehicle-treated Tg mice at 12–13 months of age was severely impaired compared with the other groups [Figure 2A, group effect: escape latency (s): *p* = 0.001; path length (cm): *p* = 0.001, latter data not shown]. This impairment was not due to motor deficits because swim speed did not differ across groups (*p* > 0.05 data not shown). Noteworthy, there was a pronounced and significant improvement in spatial learning of selenate-treated Tg mice which was comparable with the WT-groups. The comparison of vehicle- with selenate-treated Tg mice confirmed the rescue effect of chronic selenate treatment because the latter was statistically indistinguishable from the two WT groups in terms of escape latency and path length (Figure 2A). In agreement with acquisition performance, spatial retention during probe trials was significantly different between the genotypes for the target quadrant (two-way ANOVA *F*_(3,32)_ = 9.775, *p* = 0.004). However, there was also a significant effect of group detected for the opposite quadrant (*F*_(3,32)_ = 15.778, *p* < 0.001), and interaction effect (*F*_(3,32)_ = 27.388, *p* < 0.001). Dunnett tests indicated that vehicle-treated THY-Tau22 failed to develop a preference for the target quadrant in contrast to the other three groups (*p* = 0.001 for comparisons of vehicle-treated THY-Tau22 with the other three groups, Figures 2B–F). Vehicle-treated THY-Tau22 mice displayed instead a preference for the opposite quadrant (Tg_veh vs. Tg_sel *p* = 0.001, vs. wt_veh *p* = 0.002, vs. wt_sel *p* = 0.005). Analysis of the latency to cross the former platform position revealed a trend towards poorer spatial reference memory in the vehicle-treated Tg mice compared to the other groups (Two-way ANOVA *F*_(3,32)_ = 3.893, *p* = 0.057).

**Figure 3:**
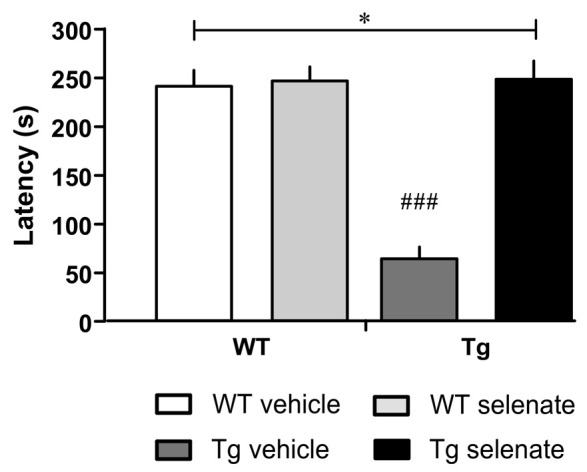
Selenate restored impaired retention memory of Tg mice in the Passive Avoidance test. Mean ± SEM is given. Group sizes: Tg-veh *n* = 9, Tg-sel *n* = 10, WT-veh *n* = 8, WT-sel *n* = 10. A bar with a * indicates *p* ≤ 0.01 in the Two-way ANOVA; ^###^ represents *p* ≤ 0.0001 in the Dunnett *post hoc* test.

Finally, in passive avoidance learning, a one-trial learning test, we found differences between the groups (overall ANOVA *F*_(3,33)_ = 32.348, *p* = 0.0001). Thus, the two WT-groups exhibited clear memory recall of the shock experienced 24 h earlier, whereas vehicle-treated Tg mice failed to remember as indicated by the short-latency to enter the dark compartment. Notably, in selenate-treated Tg animals, retention memory was restored to WT levels, resulting in a highly significant effect of treatment (Figure 3, *post hoc* Dunnett tests for vehicle-treated Tg compared to the other three groups *p* = 0.0001).

### Electrophysiological Studies

Several reports have established that chronic sodium selenate treatment rescues certain behavioral phenotypes in tau mice models, including reducing NFT load in neurons (Corcoran et al., 2010b; van Eersel et al., 2010). However, the effects of chronic selenate application on synaptic transmission and synaptic plasticity have not yet been investigated. In a recent study, we reported that transient bath-application of selenate rescued an impaired long-term depression (LTD), one of the major types of synaptic plasticity, in Tg mice *in vitro* (Ahmed et al., 2015). Here, we tested the effects of oral chronic selenate application on synaptic transmission and plasticity in the CA1-region of hippocampal slices *ex vivo* after *in vivo* treatment. At 9 months, the age at which the selenate treatment was started, Tg mice show already prominent signs of AD-like hippocampal Tau pathology as hyperphosphorylation, abnormal conformational changes, and aggregation of tau, associated with severe memory deficits and synaptic impairments. However, there are no indications of neuronal loss or neurodegeneration (Schindowski et al., 2006; Belarbi et al., 2009; Van der Jeugd et al., 2011; Burnouf et al., 2013).

**Figure 4:**
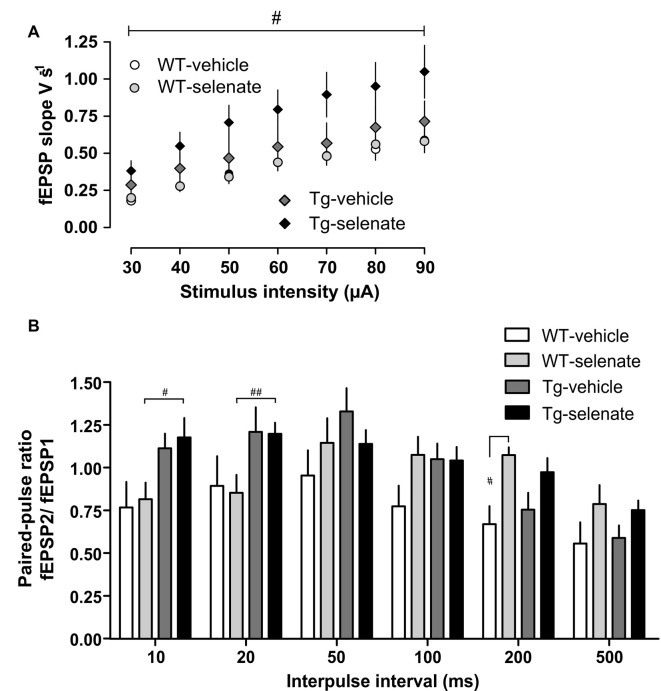
Enhanced basal synaptic transmission by selenate treatment in Tg mice. **(A)** Chronic selenate application increased basal synaptic transmission in Tg mice. Two-way ANOVA confirmed a significant between-group difference (*F*_(3,35)_ = 3.389, *p* < 0.029) and *post hoc* comparisons revealed a significant enhancement of synaptic transmission in selenate-treated Tg mice (*n* = 10) compared to selenate-treated WT mice (*n* = 8; *p* ≤ 0.020, Dunnett test with WT-sel as the control group). **(B)** Selenate-treated Tg mice (*n* = 12) had higher paired-pulse values than selenate-treated WT mice (*n* = 12) at short interpulse intervals of 10 and 20 ms. Interestingly, selenate caused a significant enhancement of paired-pulse values in WT mice at 200 ms. Two-Way ANOVA plus Dunnett *post hoc* test compared with WT-selenate. Mean ± SEM is given. Bar with ^#^indicates significance level ≤0.05 of Dunnett *post hoc* test, likewise ^#^*p* ≤ 0.05; ^##^*p* ≤ 0.01.

When basal synaptic transmission was inspected (Figure 4A), selenate treatment caused in Tg mice higher EPSP slopes in response to increasing stimulation strengths compared with the other three groups. Two-way ANOVA confirmed a significant effect of group (*F*_(3,35)_ = 3.389, *p* < 0.029) and *post hoc* Dunnett test revealed significant differences between selenate-treated WT and Tg mice (*p* ≤ 0.020). We then studied paired-pulse responses, a form of presynaptically mediated short-term plasticity. Tg mice displayed rather high paired-pulse ratios at short inter-pulse intervals of 10 ms and 20 ms independent of whether or not the animals had received selenate (Figure 4B). The increased paired-pulse values of selenate-treated Tg mice were statistically different from selenate-treated WT animals at 10 and 20 ms (10 ms: *p* = 0.0248, 20 ms: *p* = 0.0339, Tg sel *n* = 12, WT sel *n* = 12, Two-Way ANOVA plus Dunnett *post hoc* test compared with WT-selenate). Differences of similar size occurred also when the vehicle groups were compared, but the higher variability prevented statistical significance. While there were no significant differences at interpulse intervals of 50 ms and 100 ms, treatment with selenate led to significantly increased values in WT mice at 200 ms (Figure 4B, *p* = 0.0242, WT sel *n* = 12, WT veh *n* = 8, Two-Way ANOVA plus Dunnett *post hoc* comparison. A similar trend in Tg mice at 200 ms was not significant.

**Figure 5:**
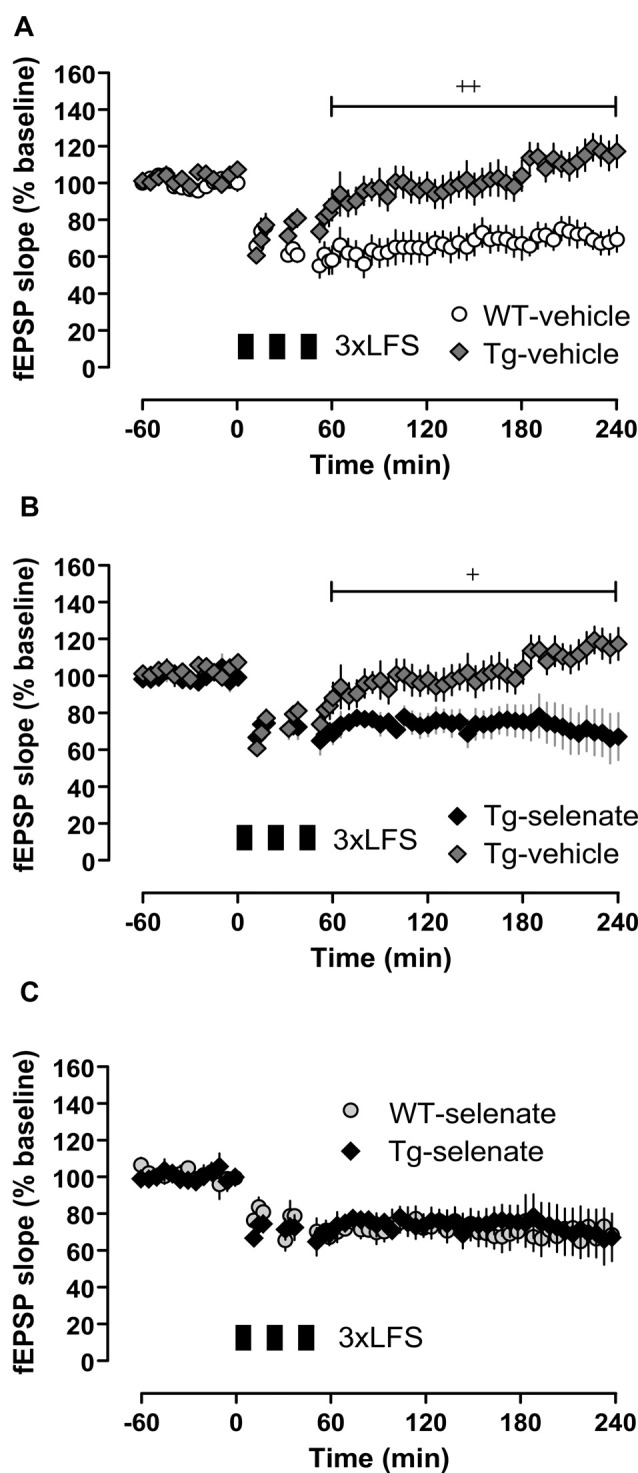
Chronic selenate treatment restored synaptic plasticity [long-term depression (LTD)] in Tg mice. RM-ANOVA of all four groups confirmed a significant main effect of group F_(2,28)_ = 6.843, *p* = 0.001. **(A)** Vehicle-treated Tg mice (*n* = 10) failed to express robust LTD which was intact in vehicle-treated WT siblings (*n* = 8). **(B)** Selenate rescued LTD in Tg (*n* = 6) compared with vehicle-treated siblings (*n* = 10). **(C)** The rescued LTD in selenate-treated Tg mice matched exactly the time-course of LTD in selenate-treated WT animals (*n* = 8). Mean ± SEM is given. Bar with cross(es) indicates a significant difference in Tukey’s *post hoc* multiple comparisons, ^+^*p* ≤ 0.05, ^++^*p* ≤ 0.01.

Next, we examined long-term synaptic plasticity. We reported earlier that the triple repetition of low-frequency stimulation (LFS) at 2 Hz was successful in inducing late-phase LTD (L-LTD) in young and middle-aged mice (Balschun et al., 2003; Van der Jeugd et al., 2011; Ahmed et al., 2015). In the current study, this robust L-LTD was expressed in WT mice but was severely impaired in Tg animals (Figure 5A). Although Tg vehicle mice attained 2 min after completion of the first LFS-train very similar values as WT vehicle animals (Tg vehicle 61 ± 3%, *n* = 10, WT vehicle 66 ± 8%, *n* = 8), the depression could not be maintained (last 30 min: Tg vehicle 117 ± 8, *n* = 10; WT-vehicle 70 ± 7%). Statistical analysis of LTD of all groups after the induction (60–240 min) with RM-ANOVA plus Tukey’s *post hoc* comparison, revealed a significant effect of group (*F*_(3,28)_ = 6.843), *p* = 0.001 and significant differences between vehicle-treated Tg animals and all other groups (Tg-sel *p* = 0.027; WT-vehicle *p* = 0.002; WT-sel 0.008). While the chronic oral application of sodium selenate had virtually no effect on WT mice, it completely rescued the L-LTD in Tg animals (Figure 5B) as convincingly demonstrated by the exact match with the LTD of the selenate-treated WT counterparts (Figure 5C).

### Immunohistochemistry and Western Blotting

We selected AT8 as the antibody of choice (Su et al., 1994; Augustinack et al., 2002; Deters et al., 2009) for the histological examination because several studies suggest that PP2Ac dephosphorylates primarily the AT8 epitopes S202/T205 while other phosphorylation sites that become hyperphosphorylated during AD, detected by antibodies such as AT100, AT180, 12E8, and PHF1, are not affected (Kins et al., 2001).

**Figure 6:**
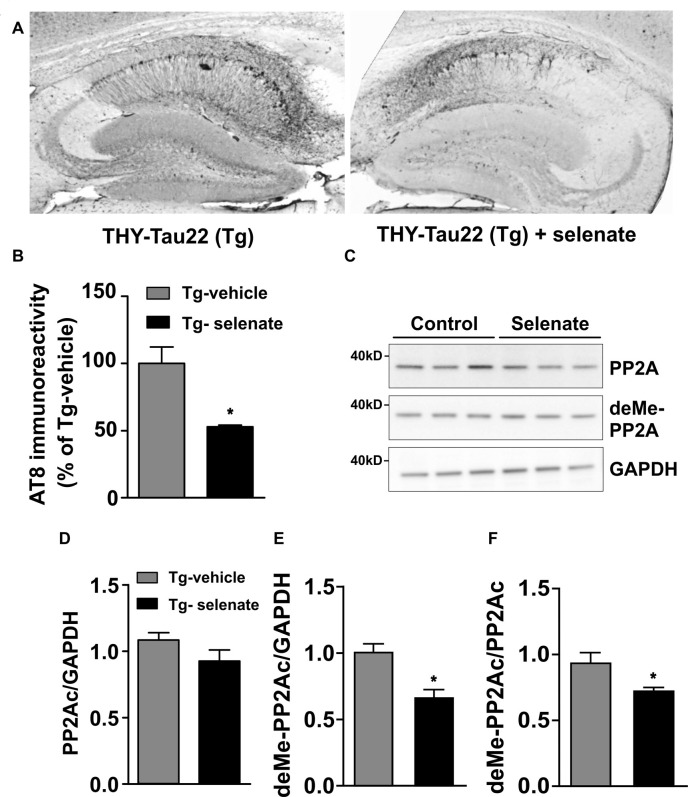
Selenate reduces AT8 immunoreactivity and antagonizes PP2A-demethylation in Tg mice. **(A)** Representative immunohistochemical sections from a vehicle-treated Tg mouse (left) and an animal that received selenate (right). Note the reduced AT8 immunoreactivity in the mouse treated with selenate. **(B)** Quantification of immunohistochemical AT8-staining from both groups. Selenate treatment reduced AT8-immunoreactivity to about 50% (*p* = 0.0027). **(C)** Immunoblots of PP2Ac and demethylated PP2Ac in vehicle-treated and selenate-treated Tg. **(D,E)** Quantification of the levels of PP2Ac and demethylated PP2Ac, respectively. The amount of demethylated PP2Ac is significantly lower after treatment with selenate, supporting higher PP2A activity under treatment. **(F)** The amount of demethylated PP2Ac (deMe-PP2Ac) normalized to the amount of PP2AC. Mean ± SEM is given. *p < 0.05, *n* = 3 per group.

Histological examination of sections from vehicle-treated (*n* = 3) and selenate-treated Tg animals (*n* = 3) with AT8, a human tau pathological marker of S202/T205 (Figure 6A), revealed a significant decrease in selenate-treated mice (*p* = 0.027, *t*-test; Figure 6B). In contrast, no significant effect was found with AT100, which recognizes Tau phosphorylation epitopes pT212/pS214 (*p* = 0.245, *t*-test, data not shown).

Since several reports have documented a downregulation of PP2Ac in murine models of tauopathies (Kins et al., 2001; Liu et al., 2005; Sontag and Sontag, 2014), we evaluated total levels of PP2Ac (normalized to GAPDH) in vehicle-treated and selenate-treated Tg mice by Western blotting (Figure 6C) and found them not being statistically different (Tg vehicle-treated: 1.08 ± 0.05, *n* = 3, Tg selenate-treated: 0.92 ± 0.08, *n* = 3, *p* = 0.174, *t*-tests; Figure 6D). When demethylation of PP2Ac (inactive PP2Ac) was quantified, a statistically significant reduction by selenate treatment was confirmed (Tg vehicle-treated: 0.93 ± 0.08, *n* = 3, Tg selenate-treated: 0.67 ± 0.05, *n* = 3, *p* = 0.049, *t*-test; Figure 6E). This difference persisted when the values of deMe-PP2Ac were normalized to the respective values of PP2Ac (Tg vehicle-treated: 0.94 ± 0.07, Tg selenate-treated: 0.732 ± 0.02, *p* = 0.042, *t*-test; Figure 6F. Interestingly, when all individual pairs of AT8 and deMePP2Ac values were subjected to correlation analysis, a significant Pearson correlation coefficient of *r* = 0.919 (*p* = 0.0096) was obtained.

## Discussion

One of the major goals of this study was to establish whether sodium selenate is a suitable candidate for an effective, mechanism-based, and inexpensive treatment to correct not only motor and behavioral phenotypes, but also synaptic deficits in a murine model of tauopathy and AD (Loef et al., 2011). The data presented here clearly demonstrate this outcome. In this study, we used male mice. It must be noted in this context, that there is an intense debate about gender effects on AD. A recent review by Nebel et al. (2018) mentions that about two-thirds of the more than 5.5 Americans afflicted with AD are women. However, a worldwide meta-analysis by Fiest et al. (2016) did not find a significantly higher prevalence of AD in women than in men after controlling for sex differences in longevity. Likewise, in two meta-analyses of worldwide studies of frontotemporal dementia, a major tauopathy, no significant gender difference in prevalence was found (Onyike and Diehl-Schmid, 2013; Hogan et al., 2016).

We found that vehicle-treated Tg mice displayed a marked increase in locomotor and exploratory activity during the initial habituation to the test environment and the dark phase of their activity cycle. This higher activity level, which was corrected by selenate treatment, is reminiscent of reports of hyperactivity in other Tau mouse models such as P301S (Przybyla et al., 2016), rTg4510 (Joly-Amado et al., 2016; Jul et al., 2016; Blackmore et al., 2017; Wang et al., 2018), MAPT-N296H (Wobst et al., 2017), N279K (Taniguchi et al., 2005) and a triple repeat mutant tau transgenic mouse line (Rockenstein et al., 2015). Of note, a Mapt^−/−^ model on a B6129PF3/J genetic background also displayed hyperactive behavior (Biundo et al., 2018). Hyperactivity was also observed in mouse models that combine Aβ- and Tau pathology such as the triple transgenic mouse line that overexpresses mutant tau *via* the taup301L transgene (Pietropaolo et al., 2008; Baeta-Corral and Gimenez-Llort, 2014; Cañete et al., 2015). As discussed in a recent review by Kosel et al. (2020), hyperactivity is a common feature of AD mouse models, including early sleep-wake disturbances and nocturnal hyperactivity. Interestingly, in a mouse model that expresses both the APP/PS1 transgene and wild-type human tau under the control of an inducible promoter, Aβ and tau were found to work cooperatively to generate hyperactive behavior (Pickett et al., 2019).

In line with other reports (Corcoran et al., 2010b; van Eersel et al., 2010; Shultz et al., 2015), chronic selenate treatment had no effects on motor control. However, we found that this compound normalized the reduced exploration of Tg in the open field as evidenced by similar values as WT controls for the number of rearings, total distance moved, and latency to enter the center of the arena. The latter could be considered as an anxiolytic effect of selenate. The difference in activity in the cage activity and the open field test can be explained by the set-up of both tests. The activity cage looks alike their home cage in the animalium: both are transparent cages composed out of the same plastic material with an identical mesh grid top and provided with the same bedding and dim-lit. In contrast, the open field arena indeed is a more anxious environment because it is composed of a non-transparent large box without bedding and brightly lit. Also, before the Open Field test, mice undergo 30 min of dark adaptation inside a dark cupboard. Taken all of this together, we, therefore, think that the mice are more anxious in the open field which is reflected in the decreased distance traveled and increased latency to enter the center of the open field the two main parameters for an anxiogenic phenotype.

We further observed that selenate treatment facilitated/rescued the contextual awareness of Tg mice in the passive avoidance test resulting in a similar performance as WT mice. Deficits in contextual hippocampus-dependent functions have been reported for several tauopathy mouse models (Fujio et al., 2007; Sydow et al., 2011; Van der Jeugd et al., 2011; Van Der Jeugd et al., 2012). The clear improvement of spatial reference memory of Tg mice in the MWM by chronic selenate treatment is corroborated by similar findings in another tauopathy mouse model (TMTH) in response to selenate (Corcoran et al., 2010b), and by marked improvements in spatial learning after treating 3xTg mice with selenomethionine (Song et al., 2014; Zhang et al., 2017), sodium selenate (Van Der Jeugd et al., 2018) and Ebselen, a lipid-soluble low molecular weight selenoorganic compound (Xie et al., 2017), respectively. However, in these studies, mitigated or rescued pathology in 3xTg mice could also be caused by dietary selenate or selenomethionine repressing amyloid-β formation and deposition *via* down-regulation of β-secretase (BACE1) expression and decreased APP cleavage (Zhang et al., 2016; Jin et al., 2017). In our study with Tg mice, rescuing effects mediated by changed amyloid-β production can be excluded.

Experimental evidence supports the activation of protein phosphatase A (PP2A) by selenate (Corcoran et al., 2010b; van Eersel et al., 2010; Brozmanova, 2011; Shultz et al., 2015; Jin et al., 2017) and selenomethionine (Zhang et al., 2017), but the precise mechanism remains unclear. Interestingly in this context, Nicholls et al. (2008) postulated a role for PP2A in cognitive tasks such as the spatial version of the MWM. In our experiments, Tg mice presented severe deficits in acquisition and probe trial performance in the MWM, i.e., under the conditions of a progressing tauopathy when PP2A activity has been described to be downregulated (Sontag and Sontag, 2014). Upon selenate treatment, we observed a restoration of spatial memory of Tg to WT-levels in the probe trials during acquisition. Thus, it appears reasonable to assume that increased PP2Ac activity can rescue or reinstate molecular processes that are important for the formation and consolidation of spatial memory.

In our electrophysiological measurements of synaptic readouts, we observed that selenate significantly increased basal synaptic transmission in Tg, as becoming overt in the input-output curves. Furthermore, we recorded increased paired-pulse ratios in Tg mice at short interpulse intervals pointing to decreased recurrent inhibition. This could be due to the loss of interneurons due to their high vulnerability during progressing AD/tau pathology as reported for several AD mouse models (Loreth et al., 2012). Such a decline in interneuron numbers is considered a major reason for the abundant occurrence of epileptic seizures in AD patients, which has been replicated in certain murine AD models (Palop and Mucke, 2016). However, in the hippocampus of Tg mice no signs of neuronal loss have been detected, but an increase in astroglial cells (Van der Jeugd et al., 2011).

Recently, Shultz et al. (2015) have reported that sodium selenate treatment increases expression of the PP2A/PR55B regulatory subunit after traumatic brain injury, arguably another tauopathy. Of note, the PP2A isoform that contains the PR55 (or PPP2R2A or Ba) regulatory subunit is the predominant neuronal PP2A holoenzyme showing the highest tau phosphatase activity and the strongest affinity for tau (Sontag et al., 1996, 1999; Xu et al., 2008). Whereas we did not measure the expression of this subunit, we compared overall changes of the catalytic subunit, PP2Ac, and found no differences between genotypes. However, when we assayed for demethylated PP2Ac (Leu309; Sontag et al., 2013), we recorded a significant decrease upon selenate treatment compared with vehicle-treated Tg, which is in agreement with published reports that methylation of the catalytic subunit PP2Ac at Leu309 by leucine carboxyl methyltransferase 1 (LCMT1) is required for enhanced PP2Ac activity and the assembly and stabilization of PP2A/Ba holoenzymes (Sontag, 2001; Sontag et al., 2004a; Sontag and Sontag, 2014). Interestingly, it has been documented that Tg mice have increased GSK3β activity (Ahmed et al., 2015) and enhanced GSK3β activity has been linked to reduced PP2Ac activity as found here (Qian et al., 2010; Yao et al., 2012).

Presynaptic mechanisms are unlikely to be included in the selenate effects as no differences in the paired-pulse ratios (PPR) at the 50 ms inter-pulse interval were found between treated and vehicle-treated Tg groups. Intriguingly, in WT mice selenate enhanced PPR at an interpulse interval of 200 ms, pointing to selenate-mediated effects on GABA_B_-mediated inhibition.

In Tg mice treated with selenate, we observed a restoration of LTD comparable with WT levels while selenate did not affect WT synaptic plasticity. This LTD rescue “phenomenon” was similar to the one that we have previously documented in response to a transient bath-application of selenate, which notably overcame okadaic acid inhibition (Ahmed et al., 2015). Further, histological evidence using the AT8 antibody, one established pathological marker for the tau phosphorylation site Ser202/Thr205 which is closely associated with PP2Ac activity (Kins et al., 2003; Deters et al., 2009), clearly identified reduced phosphorylation of this epitope after chronic selenate treatment. Recent studies have shown that the AT8 binding motif does not only include the well-known pS202/pT205 epitope but also pS208 which is important for the affinity and kinetics of AT8 binding (Malia et al., 2016). Since phosphorylation of S208 was suggested to enhance the formation of tau filaments that lead to NFTs (Xia et al., 2020), PP2Ac likely controls early hyperphosphorylation steps in tau pathogenesis that promote aggregation but are still reversible. There is also evidence from a mouse model expressing a repressible human tau variant, that the pathological processes responsible for cognitive decline and the processes underlying NFT-formation may dissociate at a certain stage of Tau pathology (SantaCruz et al., 2005).

Given the current state of knowledge, it is rather difficult to identify a certain mechanism that is causally responsible for the selenate-mediated rescue effect on synaptic plasticity and cognition. Selenate, has been used as an anti-cancer agent for many years (Corcoran et al., 2010a; Brozmanova, 2011; Rayman, 2012) and was found to be incorporated into proteins as selenocysteine. The latter is a major component of the anti-oxidant agent’s glutathione peroxidases (GP) and thioredoxin reductases (TR) which are well represented in the brain (Hoppe et al., 2008; Bellinger et al., 2009; Pitts et al., 2014). Both enzymes are greatly reduced in AD and linked to a reduction in PP2A methylation (Tolstykh et al., 2000; Vafai and Stock, 2002; Morris, 2003). Sontag et al. (2010) described a link between high levels of S-adenosylhomocysteine and a reduction in PP2A methylation levels. Moreover, PP2A methylation is under the tight control of protein phosphatase methylesterase-1 (PME1) and leucine carboxyl methyltransferase-1 (LCMT1; Sontag et al., 2010). Overexpression of PME1 in murine models had detrimental consequences on neurocognition and synaptic plasticity while overexpression of LCMT1 had beneficial effects (Nicholls et al., 2016). In AD, decreased levels of LCMT1 and methylated PP2A were described to be correlated with reduced amounts and the severity of phospho-tau pathology (Sontag et al., 2004a,b; Taleski and Sontag, 2018). Thus, changes in the amount of PP2A/Ba may lead to changes in PP2A methylation which in turn may compromise the intricate balance between the activity of tau kinases and phosphatases (Taleski and Sontag, 2018). Thus, these reports indicate that PP2A methylation is a defining factor in tauopathies.

In conclusion, we demonstrate for the first time that a 3-month treatment with dietary selenate rescues deficits in synaptic plasticity as well as learning and memory in Tg mice at an advanced stage of tauopathy, which led already to severe functional deficits at the synaptic, behavioral, and cognitive level. The completely recovered LTD demonstrates a rescue of the deleterious effects of progressive tauopathy at the level of the synapse, the main site of functional deterioration that leads to cognitive decline. This is most likely due to a reduction in the demethylated, catalytically inactive catalytic subunit PP2Ac, which shifts the equilibrium in favor of the methylated PP2Ac and thus to a higher PP2A activity. Consequently, this leads to a decrease in hyperphosphorylated, but not misconformed Tau as supported by the significant reduction in AT-8 but not in AT-100 immunostaining. Together with the clear rescuing effects on synaptic plasticity and cognitive measures in our study, this reinforces the idea that the detrimental impact of pathological Tau towards plasticity and memory is likely related to non-aggregated soluble Tau species and recommends selenate as a candidate compound for human Tau-targeting clinical trials.

## Data Availability Statement

The raw data supporting the conclusions of this article will be made available by the authors upon reasonable request.

## Ethics Statement

The animal study was reviewed and approved by the Animal experiments committee of the University of Leuven, Belgium and were carried out in accordance with the European Directive 2010/63/EU.

## Author Contributions

TA developed the idea for these studies. AVDJ and RD’H designed the behavioral experiments which were conducted by AVDJ. The electrophysiological study was designed by TA and DBa, the biochemical and histological assays by DBl and LB. TA performed the electrophysiological measurements, DBl the biochemical and histological assays. AVDJ and DBa analyzed the behavioral data, TA, and DBa the electrophysiological experiments and DBl and RC the biochemical and histological assays with input from LB. DBa, TA, and AVDJ wrote the article with contributions from DBl and LB.

## Conflict of Interest

The authors declare that the research was conducted in the absence of any commercial or financial relationships that could be construed as a potential conflict of interest.
